# 

**Published:** 1905-12

**Authors:** 


*THE BRISTOL MEDICO-CHIRURGICAL SOCIETY.*

CONTENTS.

| | PAGE |
|---|---|
| **The Family Doctor.** By JOHN DACRE, M.R.C.S. | 289 |
| **On Two Cases of Myasthenia Gravis.** By J. MICHELL CLARKE, M.A., M.D. Cantab., F.R.C.P. | 308 |
| **The Long Fox Lecture.** By R. SHINGLETON SMITH, M.D., B.Sc. Lond., F.R.C.P. | 317 |
| **Notes on the Voluntary Notification of Phthisis, and on the Municipal Attitude in Bristol in regard to the Disease.** By D. S. DAVIES, M.D. Lond. | 351 |
| **A Study of the Records of One Hundred and Fifty-five Cases of Operation for Appendicitis** *(continued).* By CHARLES A. MORTON, F.R.C.S. | 360 |
| **Progress of the Medical Sciences:** | |
| **Medicine.** J. R. CHARLES, M.A., M.D. Cantab. | 368 |
| **Surgery.** JAMES SWAIN, M.D., M.S. Lond. | 371 |
| **Reviews of Books** | 375 |
| **Editorial Notes** | 380 |
| **Notes on Preparations for the Sick** | 387 |
| **The Library of the Bristol Medico-Chirurgical Society** | 392 |
| **Meetings of Societies** | 395 |
| **Obituary Notices** | 398 |
| **Local Medical Notes** | 398 |

